# High prevalence of echocardiographic abnormalities in older HIV-infected children taking antiretroviral therapy

**DOI:** 10.1097/QAD.0000000000002031

**Published:** 2018-09-19

**Authors:** Edith D. Majonga, Andrea M. Rehman, Victoria Simms, Grace Mchugh, Hilda A. Mujuru, Kusum Nathoo, Jon O. Odland, Mohammad S. Patel, Juan P. Kaski, Rashida A. Ferrand

**Affiliations:** a London School of Hygiene and Tropical Medicine, London, United Kingdom; b Biomedical Research and Training Institute; c University of Zimbabwe, Harare, Zimbabwe; d UiT The Arctic University of Norway, Tromsø, Norway; e Department of Public Health, University of Pretoria, South Africa; f MRI & Radiology Centre, Harare, Zimbabwe; g Centre for Inherited Cardiovascular Diseases, Great Ormond Street Hospital; h Institute of Cardiovascular Science, University College London, London, United Kingdom.

**Keywords:** Africa, antiretroviral therapy, cardiac abnormalities, children, HIV

## Abstract

**Background:**

Antiretroviral therapy (ART) has decreased mortality so that increasing numbers of children with HIV are reaching adolescence. However, longstanding HIV infection and/or its treatment in children is associated with noninfectious complications including cardiac disease. We investigated the prevalence, spectrum and risk factors for echocardiographic abnormalities among children established on ART.

**Methods:**

HIV-infected children aged 6–16 years, on ART at least 6 months were enrolled into a cross-sectional study from a public-sector paediatric HIV clinic in Harare, Zimbabwe. A standardized examination including transthoracic echocardiography was performed. Local echocardiographic reference ranges were used to define cardiac abnormalities. Logistic regression was used to examine the association between cardiac abnormalities and risk factors.

**Results:**

Of the 201participants recruited, 92 (46%) were girls and median age was 11 (IQR 9–12) years; CD4^+^ cell count was 727 cells/μl (IQR 473–935) and 154 (78%) had viral load less than 400 copies/ml. Echocardiographic abnormalities were found in 83 (42%); left ventricular (LV) diastolic dysfunction was the most common abnormality 45 (23%) and LV hypertrophy in 22 (11%). LV and left atrial dilatation were found in 9 (5%) and 16 (8%), respectively. Right ventricular dilatation and systolic dysfunction were found in 13 (7%) and 4 (2%), respectively, of whom 60% had concurrent left heart abnormalities. Current use of nevirapine was associated with LVH [aOR 3.14 (1.13–8.72; *P* = 0.03)] and hypertension was associated with LV diastolic dysfunction [aOR 3.12 (1.48–6.57; *P* < 0.01)].

**Conclusion:**

HIV-infected children established on ART have a high burden of echocardiographic abnormalities. Right heart disease was predominantly associated with left heart abnormalities and may be part of a global cardiomyopathic process. Further studies are needed to investigate the natural history, aetiology, and pathogenesis of these abnormalities, so that appropriate monitoring and treatment strategies can be developed.

## Introduction

Of the approximately 3.2 million children with HIV globally, 90% live in sub-Saharan Africa [1]. The global expansion of prevention of mother-to-child HIV transmission and scale-up of antiretroviral therapy (ART) programs has resulted in a substantial decrease in the number of infants born with HIV and an increase in numbers of older children and adolescents living with HIV because of improved survival on ART [2].

Although ART has dramatically decreased the risk of opportunistic infections and mortality, there is mounting evidence that longstanding HIV infection and/or its treatment are associated with noninfectious complications, including cardiac disease [3]. Cardiac disease has been reported among treated and untreated HIV-infected adults, with prevalence ranging from 18 to 78% [4,5]. Low CD4^+^ cell count and high viral load have been identified as independent risk factors for cardiac disease, but residual confounding by well recognized traditional risk factors, such as smoking and hypertension remain an issue in these studies [5]. A small number of studies have suggested that children with HIV also are at risk of cardiac disease, despite the absence of traditional risk factors, with the most common reported abnormalities being left ventricular (LV) dilatation and systolic dysfunction [6,7]. HIV infection can also lead to right heart abnormalities, either as a consequence of pulmonary arterial hypertension (PAH) or secondary to chronic lung disease. We have previously reported a high prevalence of chronic lung disease among HIV-infected older children on ART [8,9], which may result in pulmonary hypertension and subsequent right ventricular (RV) dilatation and/or RV systolic dysfunction [10]. However, most studies were conducted in children on zidovudine monotherapy or in mixed cohorts of ART-naive and experienced participants, and were mainly performed in younger children in high-resource settings [7,11].

In the ART era, there are contrasting reports on cardiac disease in HIV-infected children who are on ART from high-resource and low-resource settings [12–14]. We, therefore, investigated the prevalence, spectrum and risk factors for cardiac abnormalities in children established on ART in Harare, Zimbabwe.

## Methods

A cross-sectional study was conducted at the paediatric HIV clinic at Harare Central Hospital, Zimbabwe, between August 2014 and June 2015. Harare Central Hospital is the largest public-sector hospital in Harare and provides HIV care to over 4000 children. This study was conducted within a larger cohort study called INHALE (Investigation of Heart and Lung Diseases in HIV among older children), aiming to investigate cardiorespiratory disease in children with HIV infection. Findings of the clinical and radiographic features of chronic lung disease have already been published [8,9]. This report is limited to echocardiographic abnormalities in the same cohort of HIV-infected children.

HIV-infected children aged 6–16 years attending for HIV care, not acutely ill and taking ART for at least 6 months, were consecutively recruited on week days, restricted to the first five eligible participants per day for logistical ease. A minimum of 6 months on ART was selected to enable sufficient time for viral suppression and for the risk of immune reconstitution syndrome to have dropped.

### Study procedures

An interviewer-administered questionnaire was used to collect sociodemographic data and clinical history, including previous illnesses, drug history and current symptoms. A standardized clinical assessment was performed, including anthropometric measurement (height and weight), heart rate, respiratory rate, blood pressure, New York Heart Association (NYHA) functional class, pulse oximetry and Medical Research Council (MRC) Dyspnoea scale. Fasted blood was collected for measurement of plasma glucose, and for HIV-1 viral load and CD4^+^ cell count testing. The CD4^+^ cell count was measured on site using an Alere PIMA analyser (Alere Technologies GmbH, Jena, Germany) and plasma viral load was measured using the COBAS protocol Ampliprep/Taqman 48 Version 2.0 (Roche Molecular System, Branchburg, New Jersey, USA). Spirometry was performed according to American Thoracic Society guidelines to assess respiratory function [15].

A transthoracic echocardiogram was performed using a Mindray DC N6 multipurpose ultrasound machine (Mindray, Shenzhen, China) by an echocardiographer trained in paediatric echocardiography (E.M.). A standard protocol consisting of two-dimensional, M-mode, pulsed and continuous wave Doppler and colour flow mapping as recommended for transthoracic echocardiography was adopted [16]. Participants were scanned in the left lateral or supine position to obtain an optimum image quality and all measurements made over three cardiac cycles. Images were acquired using a 7 MHz transducer and were saved in DICOM format for off-line analysis. Cardiac measurements were based on the American Society of Echocardiography (ASE) criteria [16]. Echocardiography scans were quality controlled by an experienced paediatric cardiologist (J.P.K.) for adequacy of views. Furthermore, a random sample of 10% scans were re-measured by an independent rater (M.S.P.) for interobserver agreement.

### Definitions

Echocardiography measures for LV and right ventricular (RV) dimensions were normalized to body surface area calculated using Du Bois and Du Bois formula [17], and converted to *z*-scores using local references [18] and further compared with European published references among Caucasian children [19]. Local reference ranges were used as the primary basis for defining cardiac abnormalities in the study. LV and RV dilatation were defined as a *z*-score greater than +2 for LV and RV diameters in diastole, respectively, left atrial dilatation was a *z*-score greater than +2 for left atrial diameter in systole and LVH was defined as maximal wall thickness of interventricular septum and/or LV posterior wall greater than +2 *z*-scores [18]. LV systolic function was assessed by Simpson's Biplane method and an ejection fraction at least 55% was considered normal [16]. LV diastolic dysfunction was assessed through transmitral Doppler [peak early (*E*) and late diastolic (*A*) filling velocities], *E*/*A* ratio, deceleration time and pulmonary venous flow velocities including peak systolic (S) and diastolic (D) waves, S/D ratio and atrial reversal (Ar) velocity and paediatric reference ranges were used to define abnormality [20]. Patients were classified as having diastolic dysfunction when at least four parameters were abnormal [21]. RV systolic dysfunction was defined as a tricuspid annular plane systolic excursion (TAPSE) *z*-score of less than −2 [18]. Pulmonary hypertension was defined as present if the tricuspid regurgitation velocity was at least 2.9 m/s, estimated pulmonary arterial systolic pressure (PASP) at least 37 mmHg with/without additional echocardiographic variables suggestive of pulmonary hypertension (assuming right atrial pressure of 5 mmHg) [22]. PASP was indirectly calculated from the pressure gradient across the tricuspid valve by measuring the regurgitant jet and applying the simplified Bernoulli equation (*4V*2) and adding right atrial pressure (RAP) estimate to the tricuspid pressure gradient [22]. Hypoxia was defined as a resting oxygen saturation less than 88% or a at least 5% desaturation immediately following exercise; resting tachypnea was defined as a respiratory rate less than 25/min. Sinus tachycardia was defined as less than 130 beats per minute (bpm) for children aged 6–8 years and greater than 110 bpm for those aged 9–16 years [23]. Stunting and wasting were defined as a *z*-score less than −2 for height-for-age and weight-for-age, respectively, using British 1990 growth references [24]. Hypertension was defined as the SBP and/or DBP at least 95th percentile; prehypertension was SBP and/or DBP between the 90th and 95th percentile for age, sex and height [25]. Chronic lung disease was defined as having at least one of the following criteria: chronic cough (≥3 months) with tuberculosis excluded; hypoxia (SpO_2_ <90% or desaturation ≥5% upon exertion); abnormal spirometry (defined as reduced ratio of the highest forced expiratory volume in 1 s (FEV1) and forced vital capacity (FVC; FEV1 : FVC) or reduced FVC regardless of normal FEV1 : FVC ratio) irreversible with salbutamol [9] and MRC Dyspnoea scale greater than 1 [26].

### Data management and statistical analysis

Data were extracted from paper forms using optical character recognition software (Cardiff TELEFORM Intelligent Character, Version 10.7). Data were analyzed using STATA version 12 software (StataCorp, College Station, Texas, USA).

A previous, retrospective, cross-sectional study in Zimbabwean adolescents found the prevalence of cardiac abnormalities to be between 24 and 67% [27]. We calculated a sample size of 200 was required to estimate an assumed prevalence of 65% with a precision of +/−8 to 10%, for a 95% confidence interval. The association between HIV-related and clinical factors, determined a priori, and left and right cardiac abnormalities was investigated using multivariate logistic regression and odds ratios were calculated. Age and sex were included as a priori variables. HIV-related factors were categorized as follows: CD4^+^ cell count (>200 cells/μl and ≤200 cells/μl), HIV viral load (≤400 copies/ml and >400 copies/ml), age at ART initiation (0–5, 6–10 and 11–16 years), duration on ART (≤2 years and >2 years) and age was categorized as 6–10 years and 11–16 years. Antiretroviral drugs, cardiac signs and symptoms, hypertension and chronic lung disease were dichotomized into ‘yes’ and ‘no.’ Zidovudine and nevirapine were selected because of the evidence that zidovudine is associated with development of cardiomyopathy in children and nevirapine is associated with LVH in adults [28,29]. Clinical factors evaluated include cardiac signs and symptoms, hypertension and chronic lung disease. Cardiac signs and symptoms included hypoxia, chest pains, tachypnoea and ankle swelling, and were grouped into one variable called cardiac symptoms. HIV-related variables that were significant at *P* ≤ 0.1 on univariate logistic regression analysis were included in a multivariate logistic regression model. Clinical variables were added to the model and any clinical variable that was significant at *P* ≤ 0.1 was retained for inclusion in the final model. A value of *P* ≤ 0.05 was considered statistically significant in the final model. Intraobserver and interobserver agreements were assessed through Bland–Altman plots [30]. Variability was estimated by calculating the mean (95% CI) of the arithmetic differences between repeated cardiac measures of the same participant. Normally distributed differences would fall within a range of mean ± 1.96 standard deviation (SD) and the range is referred to as limits of agreement (LoA).

Ethical approval was obtained from the Medical Research Council of Zimbabwe, the London School of Hygiene and Tropical Medicine Ethics Committee, the Biomedical Research and Training Institute Institutional Review Board and the Harare Central Hospital Ethics Committee. Written informed consent from guardians and assent from participants was obtained prior to enrolment. Any abnormal findings during the course of the study were recorded in the child's notes and the child was referred to a clinician on the same day for further management.

## Results

### Clinical characteristics

**Fig. 1:**
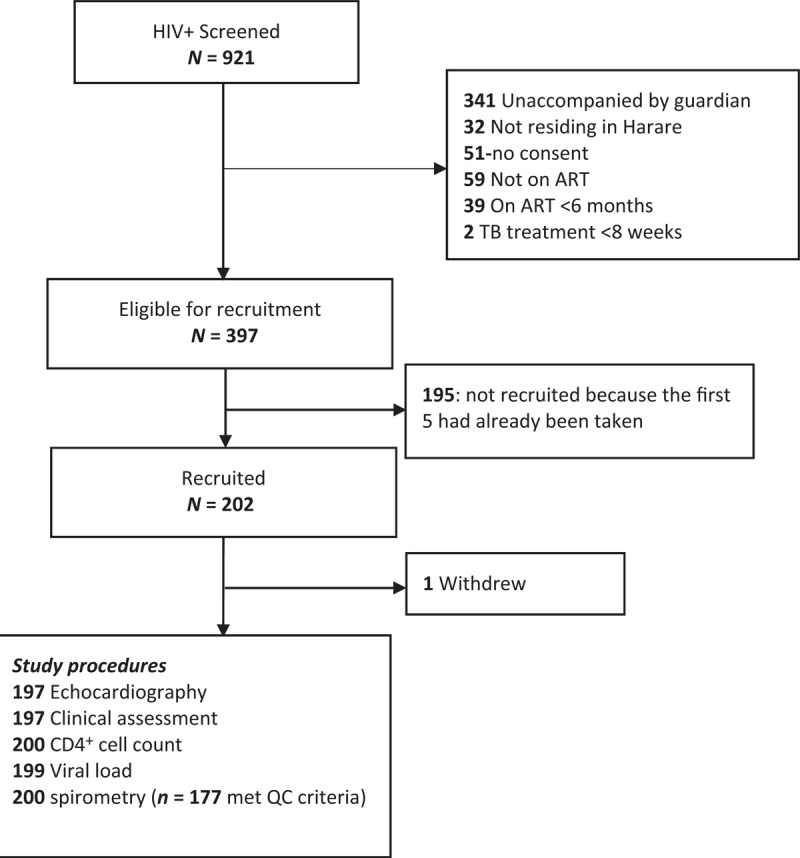
Participant recruitment.

Of the 921 attendees to the clinic aged 6–16 years over the study period, 397 were eligible and of these, 201 children were enrolled; the remainder were excluded because of the total number of enrolments being restricted to the first five eligible attendees per day (Fig. 1). The median age was 11.1 years (IQR, 9–12) and 92 (46%) were girls. Mother-to-child transmission was assessed as the likely mode of HIV acquisition in all but one participant, in whom we speculate that HIV had been acquired through sexual transmission because the mother had tested HIV negative when the child was diagnosed at the age of 6 years. Participants were taking ART for a median duration of 4.7 years (IQR, 2.6–6.4) and 154/197 (78%) had an HIV viral load less than 400 copies/ml; the median CD4^+^ cell count at enrolment was 727 cell/μl (IQR, 473–935) (*n* = 198). No participant was taking any medication for cardiac disease. Eligible children who were not enrolled in the study were on average 5 months younger than those enrolled and had initiated ART at a year younger (Supplementary Table 1).

Thirty-four (17%) children had a history of chest pain, 36 of 201 (18%) were in NYHA functional class greater than 1 and 24 of 198 (12%) had hypoxia. An abnormal blood pressure was observed in 106 of 198 (54%) children: 85 of 106 (80%) had hypertension and 21 of 106 (20%) had prehypertension. Chronic cough was reported in 30 (15%) and dyspnoea using the MRC dyspnoea scale in 30 (15%) and 42 (24%) had abnormal lung function on spirometry (Table 1).

### Echocardiographic findings

Of the 201 enrolled participants, echocardiograms were performed on 197; the remaining four did not return for the examination. Echocardiographic measures are summarized in Table 2. Eighty-three (42%) participants had an echocardiographic abnormality of either the left and/or right heart. The most common abnormal finding was LV diastolic dysfunction in 45 of 197 (23%) children (Table 3). Isolated diastolic dysfunction, without associated LV dilatation or LVH, was observed in 33 of 45 (73%) children. LVH was the second most common finding in 22 of 197 (11%) with the following patterns: interventricular septal hypertrophy two of 22 (9%) and posterior wall hypertrophy 20 of 22 (91%). Seven children (32%) with LVH had diastolic dysfunction and global systolic function was impaired in one child. LV dilatation was observed in nine of 197 (5%) children, one of whom had impaired systolic function and pericardial effusion. Left atrial dilatation was found in 16 of 197 (8%) children and of these four (25%) had LVH; four (25%) had diastolic dysfunction and two (12%) had LV dilatation.

RV dilatation was found in 13 of 197 (7%) and RV systolic dysfunction in four of 180 (2%) including two children with both RV dilatation and systolic dysfunctions. Of the 15 participants who had RV abnormalities, nine (60%) had concurrent left heart abnormalities, including isolated LVH (*n* = 2), isolated LV dilatation (*n* = 1), isolated left atrial dilatation (*n* = 1), LV systolic dysfunction and LVH (*n* = 1), isolated LV diastolic dysfunction (*n* = 3) and one participant who had a dilated cardiomyopathy with LV and left atrial dilatation and LV systolic dysfunction. Five (33%) of the participants with right heart abnormalities also met the case definition for chronic lung disease. None of the participants met the echocardiographic criteria for pulmonary hypertension. The prevalence of echocardiographic abnormalities was much higher when European reference ranges were used (Table 3).

Twenty (10%) echocardiograms were used to determine inter-observer agreement. Bland–Altman plots showed that there was good agreement for repeated measures by the same observer 2 weeks apart and repeated measures between the two observers (Figure S1).

### Factors associated with echocardiographic abnormalities

LV diastolic dysfunction was associated with hypertension [aOR 3.12 (1.48–6.57; *P* < 0.01)]. Hypertension and LV diastolic dysfunction were present in 33 of 45 (73%) of the participants. LVH was associated with current use of nevirapine [aOR 3.14 (1.13–8.72; *P* = 0.03)] (Table 4). No HIV-related factors nor symptoms were associated with left atrial dilation. No associations were found between RV abnormalities and HIV-related factors including CD4^+^ cell count, viral load, duration on ART, age at ART initiation and lung function (supplementary Table 2).

## Discussion

The current study demonstrates a high prevalence of echocardiographic abnormalities among HIV-infected children established on ART, more than three-quarters of whom were virologically suppressed. Most of the participants with diastolic dysfunction had preserved systolic function. LV diastolic dysfunction is well described in adults with HIV but there are limited data on diastolic dysfunction in children. Diastolic parameters are difficult to measure in children and very slight alterations can result in significant changes in interpretation of the diastolic function. Interestingly, in the pre-ART era, systolic dysfunction and LV dilatation rather than impaired LV diastolic function were the predominant abnormalities observed, suggesting that LV diastolic impairment may be an earlier manifestation of cardiac disease [31]. It is not uncommon for diastolic dysfunction to present in isolation, typically before systolic impairment becomes apparent, for example, in ischaemic cardiomyopathy [32].

Among those with LVH, LV posterior wall thickening was predominant, in keeping with our previous report [27]. Idris *et al.* found that children with HIV and exposed to ART had thicker LV posterior walls compared with ART-naive children and larger LV dimensions compared with uninfected children [33]. LV dilatation was less common than LVH in our study. We found that current use of nevirapine was associated with LVH, which may suggest possible treatment induced effect on the myocardium. Nevirapine has been associated with LVH among HIV-infected adults in Spain, most of whom were on ART and virally suppressed [28].

An important, previously unreported, abnormality found in our study was left atrial dilatation, which is associated with adverse clinical outcomes such as atrial fibrillation and all-cause mortality in a range of cardiac disease, including ischaemic heart disease and hypertrophic cardiomyopathy [34]. Left atrial dilatation may occur because of increased LV filling pressures in the context of impaired LV diastolic function, for example, among those with LVH, or because of LV dilatation [34]. Our data suggest that, in this population, left atrial dilatation is related to the former. Further studies are required to investigate whether left atrial dilatation also represents a marker of increased risk of mortality among individuals living with HIV.

More than half of the children had strikingly high blood pressure, but no association was found between LVH and abnormal blood pressure. However, we did find that LV diastolic dysfunction was associated with elevated blood pressure; hypertension is an established risk factor for LV diastolic dysfunction and a major contributor of heart disease [35]. However, not all patients with diastolic dysfunction had hypertension, suggesting a possible additional primary myocardial aetiology. Chatterton-Kirchmeier *et al.*[36] also reported a high prevalence of elevated blood pressure among HIV-infected children and adolescents (age range 2–17 years), most of whom were on ART. The reasons for this remain unclear, but this finding merits further investigation in this population. A high prevalence of elevated blood pressure in African children has been previously reported, in keeping with the present study [37–40]. There is a possibility of misclassification of participants as hypertensive; although we used the Fourth Report on the Diagnosis, Evaluation, and Treatment of High Blood Pressure in Children and Adolescents, we did not perform repeated measures of BP for our participants on separate visits [25]. Furthermore, there may have been an element of ‘white-coat hypertension.’ Another explanation could be the possibility that the definition used for hypertension in this study, which is derived from reference ranges obtained from 70 000 children and adolescents from USA, is not suitable for the population under study [25]. Currently, there are no blood pressure references for African children.

We found RV abnormalities (dilatation and systolic dysfunction) in 8% of the children. RV dilatation was the most common right sided abnormality, although lower than the 29% previously reported among Zimbabwean perinatally HIV-infected adolescents [27]. The former study enrolled older children aged between 10 and 19 years and included both ART-naive and ART-experienced children. Chelo and colleagues reported an even higher prevalence of RV dilatation (76%) among Cameroonian children aged 1–15 years, 91% of whom were on ART. The Zimbabwean study used European children's references by Kampmann *et al.*[19], and the Cameroonian study used adults’ references as recommended by the ASE [41], and it is possible that the prevalence of RV dilatation may have been overestimated in both studies. A comparison of African reference ranges, derived from Zimbabwean HIV-uninfected children and the European references by Kampmann *et al.*[19] and Huwez *et al.*[42] showed that RV diameters are higher among Black African children [18]. In this study, we used locally developed reference ranges as a control population to define echocardiographic abnormalities [18]. European echocardiographic references derived from Caucasians [19] over-estimated the prevalence of some abnormalities in our cohort, underscoring the importance of using local reference ranges. Western reference ranges may not be appropriate and may explain the differences in prevalence of cardiac disease with the present study.

We did not find any association between RV abnormalities and chronic lung disease or other HIV-related factors and this may be because of a lack of power to detect the associations. More than half of the children with RV abnormalities had left heart abnormalities too, suggesting that the abnormalities in the right heart may be part of a global cardiomyopathic process. RV impairment may be secondary to left-sided cardiomyopathy and several mechanisms have been postulated, including the possibility that the same cardiomyopathic process may affect both ventricles. Alternatively, LV failure may result in reduced coronary perfusion for both LV and RV, or the dilated LV may cause RV diastolic dysfunction because of cumulative pericardial limitation [43].

Most of the children in our study had normal RV systolic function despite having RV dilatation. Ventricular dilatation commonly occurs as an early structural change to maintain stroke volume when there is reduced wall motion [44]. It is possible that ventricular systolic dysfunction was subclinical and would clinically manifest over time [45].

None of the children had elevated PASP in our study, using Doppler echocardiography. Although Doppler echocardiography is recognized as an important tool for screening and assessment of patients at risk of pulmonary hypertension [46], right heart catheterization is the gold standard in the diagnosis of pulmonary hypertension, and recent reports have shown that Doppler echocardiography may underestimate or overestimate pulmonary pressures, especially in children with elevated right heart pressures [47]. It is possible, therefore, that right heart pressures may have been underestimated in our study. However, the concurrent right and left heart abnormalities in some of the children suggest that the observed RV abnormalities are not related to undiagnosed pulmonary hypertension but, rather, may reflect an underlying biventricular cardiomyopathic process. Further studies are required to investigate this.

Although one-fifth of the children reported one or more cardiac symptoms, most children with cardiac abnormalities were asymptomatic. These findings highlight the importance of regular cardiac screening in this population, even in the absence of symptoms. Myocardial disease is often subclinical and may only become symptomatic once it progresses and leads to significant cardiac dysfunction. Relying on symptoms alone without echocardiography screening may, therefore, result in delayed diagnosis of cardiac disease.

Pathogenesis of cardiac disease in HIV infection is likely multifactorial. HIV causes dysregulated systemic activation, which leads to chronic inflammation. These mechanisms contribute to organ damage. Furthermore, damage to the immune system before subsequent access to ART maybe responsible for this long-term effect of cardiac abnormalities. Although the children in this study were stable on ART with high CD4^+^ cell count and virally suppressed at the time of the study, low nadir CD4^+^ cell count and/or opportunistic infections may have occurred and contributed to cardiac damage. Cardiotropic viruses including cytomegalovirus, coxsackievirus and Epstein–Barr virus have been reported to cause cardiac dysfunction in HIV [48]; although we did not investigate presence of these viruses in these children. Deficiency of trace elements such as selenium have also been associated with HIV-associated cardiomyopathy [49], but were not measured in this study.

A major strength of this study was the systematic cardiological assessment, with echocardiography performed prospectively by a pediatric echocardiographer. Most importantly, we used local echocardiography reference ranges to define cardiac abnormalities [18]. Participants were not recruited selectively based on symptoms. Our study is limited by lack of data on global LV longitudinal strain, which may have been more sensitive to detecting subclinical LV systolic dysfunction, not apparent in the form of reduced LV ejection fraction. It is also limited by lack of a control group, but all cardiac abnormalities were defined using reference measures derived locally. Local reference ranges were, however, not available for blood pressure. Hyperlipidemia is a recognized risk factor for cardiac disease, but we were unable to assess lipid profiles because of resource constraints. The children in this study did not undergo routine blood haemoglobin testing, although there was no clinical suspicion of anaemia. The study is cross-sectional and may have been underpowered to detect any associations between risk factors and cardiac abnormalities. Due to the cross-sectional design, no causality can be attributed to the factors that were associated with cardiac abnormalities.

In conclusion, our study demonstrates that there is a high burden of echocardiographic abnormalities in children, despite good control of HIV infection with ART. Our findings also suggest that right heart abnormalities in HIV-infected children on ART appear to be associated with abnormalities of the left heart. The impact and clinical course of these abnormalities and potential for reversibility still need to be investigated in prospective longitudinal studies. Further study of the pathogenesis of cardiac abnormalities will inform development of appropriate screening and therapeutic strategies for an increasing number of children growing up with HIV who face the prospect of taking lifelong ART.

## Acknowledgements

We would like to thank Harare Children's Hospital, the clinic staff, participants and their families.

Funding: This work was funded by the Wellcome Trust (095878/Z/11/Z). Salary support for A.M.R. was provided by the UK Medical Research Council through a grant to the LSHTM Tropical Epidemiology Group; grant code MR/K012126/1.

J.P.K. is a member of the European Reference Network on Heart Diseases (ERN GUARD-HEART).

### Conflicts of interest

There are no conflicts of interest.

## Supplementary Material

Supplemental Digital Content

## Figures and Tables

**Table 1:** Baseline characteristics of participants (*n* = 201).

Variable	N (%) unless otherwise stated
Female	92 (46)
Age (years) (median, IQR)	11.1 (9–12)
Age at HIV diagnosis, years (median, IQR)	5.1 (3-7)
CD4^+^ cell count (cells/μl) (median, IQR)	726 (473–935)
Viral load less than 400 copies/ml	154 (78)
Duration on ART (years; median, IQR)	4.7 (2.6–6.4)
Age at ART initiation (years; median, IQR)	6 (3–8)
Treated for TB	74 (37)
ART regimen	
Two NRTIs with PI (*n*)	40 (19.9)
Two NRTIs with NNRTI (*n*)	154 (76.6)
Unknown (*n*)	7 (3.5)
Antiretroviral drugs^d^	
Zidovudine	105 (52)
Nevirapine	103 (51)
Symptoms and signs	
Chest pains on exertion	34 (17)
Dizziness	19 (9)
Tachycardia at rest	13 (6.5)
Tachypnoea	14 (7.2)
Hypoxia^a^	24 (12)
Chronic cough	30 (15)
Abnormal spirometry	42 (24)
Ankle swelling	4 (2)
Wasting	44 (22)
Stunting	48 (24)
NYHA functional class >1	36 (18)
Abnormal blood pressure^b^	106 (54)
Prehypertension	*21 (20)*
Hypertension	*85 (80)*
High fasting glucose (>7)^c^	2 (1)

ART, antiretroviral therapy; NRTI, nucleoside reverse transcriptase; NNRTI, nonnucleoside reverse transcriptase; NYHA, New York heart association; IQR, interquartile range. The values in italics are sub-categories of abnormal blood pressure. The denominator used in the sub-categories is 106 (from the abnormal blood pressure).

^a^Missing data on *n* = 3.

^b^Missing data on *n* = 4.

^c^missing data on n = 7.

^d^Antiretroviral drugs evaluated in the logistic regression model.

**Table 2:** Echocardiographic measures.

Measurement	*N* = 197 [median (IQR)]
Body surface area (m^2^)	1.03 (0.92–1.15)
LV diameter *z*-score	0.68 (−0.22–1.25)
IVS diameter i-score	0.09 (0.66–0.80)
LV posterior wall *z*-score	0.28 (−0.51–1.07)
Left atrium diameter *z*-score	0.32 (−0.44–1.20)
Fractional shortening (%)	31 (5.2)^a^
Ejection fraction (%)	62 (6.6)^a^
*E* wave (m/s)	0.91 (0.81–1.02)
*A* wave (m/s)	0.53 (0.47–0.60)
*E*/*A* ratio	1.70 (1.50–1.99)
Deceleration time (ms)	173 (156–190)
PV *S* wave (m/s)	0.49 (0.41–0.56)
PV *D* wave (m/s)	0.50 (0.46–0.57)
PV *A* wave (m/s)	0.18 (0.16–0.21)
PV *S*/*D* ratio	0.96 (0.79–1.16)
RV diameter, *z*-score	0.37 (−0.52–1.28)
PASP (mmHg)	12.8 (8.7–16.9)
TAPSE, *z*-score	−0.73 (−1.44– −0.09)

D, diastolic; E/A ratio, mitral valve peak early to late left ventricular filling velocity; IVS, interventricular septum, LV, left ventricle; PASP, pulmonary arterial systolic pressure; PV, pulmonary venous; S, systolic; TAPSE, tricuspid annular plane systolic excursion.

^a^Mean (SD).

**Table 3:** Proportions of cardiac abnormalities.

Abnormalities	Local references^a^ [18] *N* = 197; *N* (%)	European references [19] *N* = 197; *N* (%)
LV dilatation	9 (5)	9 (5)
LVH	22 (11)	73 (37)
Interventricular septal hypertrophy	2 (9)	52 (71)
Posterior wall hypertrophy	20 (91)	4 (6)
Concentric hypertrophy	−	17 (23)
Left atrial dilatation	16 (8)	12 (6)
LV systolic dysfunction	3 (2)	3 (2)
LV diastolic dysfunction	45 (23)	45 (23)
RV dilatation	13 (7)	57 (29)
RV systolic dysfunction	4 (2)	−
Any echocardiographic abnormality	83 (42)	
Any left heart abnormality	77 (39)	
Any right heart abnormality	15 (8)	

LA, left atrium; LV, left ventricle; LVH, left ventricular hypertrophy; RV, right ventricular. Missing data on *n* = 4.

^a^Local reference ranges were used as the primary basis of defining cardiac abnormalities.

**Table 4:** Factors associated with left heart abnormalities.

		LV diastolic dysfunction				LVH				
Variable	Prevalence	Unadjusted		Adjusted		Prevalence	Unadjusted		Adjusted	
	*n/N* (%)	OR (95% CI)	*P* value	OR (95% CI)	*P* value	*n*/*N* (%)	OR (95% CI)	*P* value	OR (95% CI)	*P* value
Sex										
Female	20/94 (21	1				14/94 (15)	1			
Male	25/103 (24)	0.19 (0.16–2.31)	0.61			8/103 (8)	0.48 (0.12–1.21)	0.12		
Age										
6–10 years	25/91 (27)	1				10/91 (11)	1			
11–16 years	20/106 (19)	0.61 (0.31–1.20)	0.15			12/106 (11)	1.03 (0.42–2.52)	0.94		
Age at ART initiation										
0–5 years	27/93 (29)	1.91 (0.93–3.90)	0.08	1.95 (0.93–4.07)	0.08	13/93 (14)	2.14 (0.77–5.91)	0.14		
6–10 years	15/85 (18)	1		1		6/85 (7)	1			
11–16 years	3/17 (18)	1.00 (0.26–3.92)	1	1.14 (0.28–4.64)		3/17 (18)	2.82 (0.63–12.6)	0.18		
Duration on ART										
≤ 2 years	10/55 (18)	1				6/55 (11)	1			
>2 years	35/142 (25)	1.47 (0.67–3.22)	0.33			16/142 (11)	1.04 (0.38–2.80)	0.94		
CD4^+^ cell count										
>200 cells/μl	44/187 (24)	1				21/187 (11)	1			
≤200 cells/μl	1/9 (11)	0.41 (0.05–3.34)	0.40			1/9 (11)	0.99 (0.12–8.30)	0.99		
Viral load										
≤400 copies/ml	35/152 (23)	1				18/152 (12)	1			
>400 copies/ml	9/41 (22)	1.06 (0.46–2.44)	0.88			3/41 (7)	1.70 (0.48–6.08)	0.41		
Nevirapine^a^										
No	22/98 (22)	1				6/98 (6)	1			
Yes	23/99 (23)	1.05 (0.54–2.03)	0.90			16/99 (16)	2.96 (1.10–7.91)	0.03	3.14 (1.13–8.72)	0.03
Zidovudine^a^										
No	17/95 (18)	1				8/95 (8)	1			
Yes	28/102 (27)	1.74 (0.89–3.43)	0.11			14/102 (14)	1.73 (0.69–4.33)	0.242		
Cardiac symptoms^b^										
No	27/119 (23)	1				13/119 (10)	1			
Yes	18/78 (23)	1.02 (0.52–2.02)	0.95			9/78 (12)	1.06 (0.43–2.62)	0.89		
Hypertension										
No	12/92 (13)	1				12/92 (13)	1			
Yes	33/103 (32)	3.14 (1.51–6.55)	<0.01	3.12 (1.48–6.57)	<0.01	10/103 (10)	0.72 (0.29–1.75)	0.46		

ART, antiretroviral therapy; LV, left ventricular; LVH, left ventricular hypertrophy.

^a^Antiretroviral drugs.

^b^Cardiac signs and symptoms included hypoxia, chest pains, tachypnoea, and ankle swelling.
