# Supplementary material for: High prevalence of echocardiographic abnormalities in older HIV-infected children taking antiretroviral therapy
Source: AIDS. 2018 Sep 19;32(18):2739–48. doi: 10.1097/QAD.0000000000002031 (PMC6250247; doi:10.1097/QAD.0000000000002031)
Supplement: Supplemental Digital Content [file aids-32-2739-s001.pdf]

Supplementary Figure 1: Bland-Altman plots for intra-and interobserver agreement for cardiac measures

A: Intra-observer agreement Bland-Altman plots

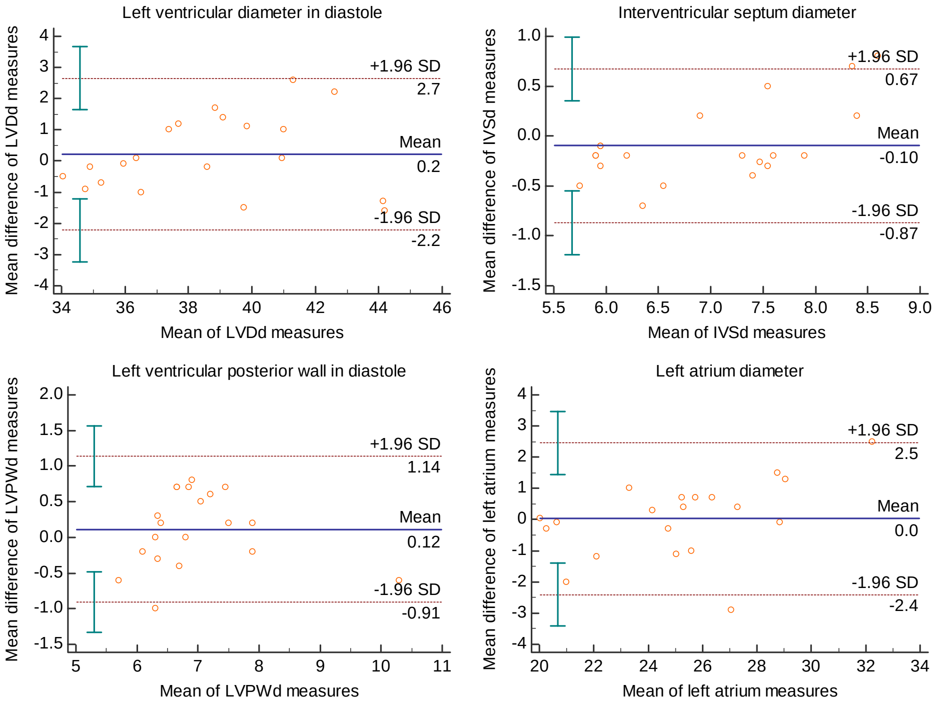

B: Inter-observer agreement plots

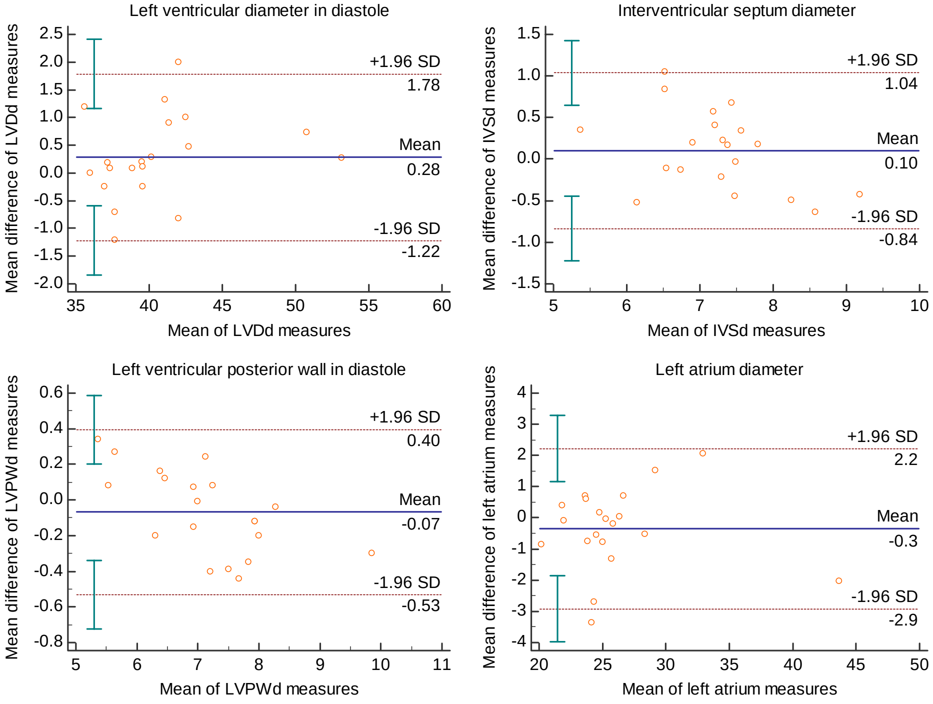

Supplementary Table 1: Factors associated with enrolment in the study

Variable	Enrolled N = 200	Not enrolled N = 197	Test coefficient, p-value
Age, y, mean (SD)	10.5 (2.5)	9.9 (2.7)	t = -2.20, p = 0.03
Sex, n (%) female	96 (48.0)	86 (43.7)	Chi^2 = 0.75, p = 0.39
Taking TB treatment, n (%)	1 (0.5)	1 (0.5)	Chi^2 = 0.00, p = 0.99
Age at ART initiation, y, mean (SD)	6.1 (3.2)	5.1 (3.3)	t = -3.04, p = 0.003

SD, standard deviation; TB, tuberculosis; ART, antiretroviral therapy

Supplementary Table 2: Factors associated with RV abnormalities

Variable	**RV abnormalities**		
	Prevalence	**Unadjusted**	
	**n/N (%)**	**OR (95% CI)**	**P-value**
**Sex**			
Female	9/94 (9)	1	
Male	6/103 (5)	0.58 (0.20- 1.71)	0.33
**Age**			
6–10 years	6/91 (7)	1	
11–16 years	9/106 (8)	1.31 (0.45- 3.84)	0.50
**Age at ART initiation**			
0–5 years	6/93 (6)	0.77 (0.25–2.38)	0.65
6–10 years	7/85 (8)	1	
11–16 years	2/17 (12)	1.49 (0.28–7.86)	0.64
**Duration on ART**			
≤ 2 years	5/55 (9)	1	
>2 years	10/142 (7)	0.80 (0.28- 2.30)	0.6
**CD4 count**			
>200 cell/µl	14/187 (7)	1	
≤200 cell/µl	0	-	-
**Viral load**			
≤400 copies/ml	15/152 (10)	1	
>400 copies/ml	0	-	-
**Nevirapine ***			
No	9/98 (9)	1	
Yes	6/99 (6)	0.64 (0.22–1.87)	0.41

**Zidovudine***			
No	8/95 (8)	1	
Yes	7/102 (7)	0.80 (0.28–2.30)	0.68
**Cardiac symptoms**†			
No	9/119 (8)	1	
Yes	6/78 (8)	1.02 (0.35–2.98)	0.97
**Hypertension**			
No	8/92 (9)	1	
Yes	7/103 (7)	0.77 (0.27–2.20)	0.62
**Chronic lung disease**			
No	10/121 (8)	1	
Yes	5/76 (7)	0.78 (0.26–2.38)	0.67

*ART, antiretroviral therapy; RV, right ventricular; * Antiretroviral drugs; † cardiac signs and symptoms included hypoxia, chest pains, tachypnoea, and ankle swelling*
